# Prevalence and clinical associations of wheezes and crackles in the general population: the Tromsø study

**DOI:** 10.1186/s12890-019-0928-1

**Published:** 2019-09-11

**Authors:** J. C. Aviles-Solis, C. Jácome, A. Davidsen, R. Einarsen, S. Vanbelle, H. Pasterkamp, H. Melbye

**Affiliations:** 1 General Practice Research Unit, Department of Community Medicine, UIT the Arctic University of Norway, Tromsø, Norway; 2 CINTESIS - Center for Health Technology and Services Research, Faculty of Medicine, University of Porto, Porto, Portugal; 3 Department of methodology and statistics, University of Maastricht, Maastricht, The Netherlands; 4 Department of Pediatrics and Child Health, University of Manitoba, Winnipeg, Canada

**Keywords:** Wheezes, Crackles, Auscultation, Population

## Abstract

**Background:**

Wheezes and crackles are well-known signs of lung diseases, but can also be heard in apparently healthy adults. However, their prevalence in a general population has been sparsely described. The objective of this study was to determine the prevalence of wheezes and crackles in a large general adult population and explore associations with self-reported disease, smoking status and lung function.

**Methods:**

We recorded lung sounds in 4033 individuals 40 years or older and collected information on self-reported disease. Pulse oximetry and spirometry were carried out. We estimated age-standardized prevalence of wheezes and crackles and associations between wheezes and crackles and variables of interest were analyzed with univariable and multivariable logistic regressions.

**Results:**

Twenty-eight percent of individuals had wheezes or crackles. The age-standardized prevalence of wheezes was 18.6% in women and 15.3% in men, and of crackles, 10.8 and 9.4%, respectively. Wheezes were mostly found during expiration and crackles during inspiration. Significant predictors of expiratory wheezes in multivariable analyses were age (10 years increase - OR 1.18, 95%CI 1.09–1.30), female gender (1.45, 1.2–1.8), self-reported asthma (1.36, 1.00–1.83), and current smoking (1.70, 1.28–2.23). The most important predictors of inspiratory crackles were age (1.76, 1.57–1.99), current smoking, (1.94, 1.40–2.69), mMRC **≥**2 (1.79, 1.18–2.65), SpO_2_ (0.88, 0.81–0.96), and FEV_1_ Z-score (0.86, 0.77–0.95).

**Conclusions:**

Nearly over a quarter of adults present adventitious lung sounds on auscultation. Age was the most important predictor of adventitious sounds, particularly crackles. The adventitious sounds were also associated with self-reported disease, current smoking and measures of lung function. The presence of findings in two or more auscultation sites was associated with a higher risk of decreased lung function than solitary findings.

**Electronic supplementary material:**

The online version of this article (10.1186/s12890-019-0928-1) contains supplementary material, which is available to authorized users.

## Background

Two hundred years after its invention, the relevance of the stethoscope in modern medical practice has become a topic of debate [1, 2]. There are some obvious advantages of lung auscultation, such as availability, low cost and non-invasiveness. Lung auscultation remains thus an important part of the respiratory examination, mainly in primary care and in resource-constrained settings.

Lung auscultation has shown to be useful in diagnosing various respiratory disorders. Adventitious lung sounds (ALS) such as wheezes and crackles are associated with common diseases like asthma [3], chronic obstructive pulmonary disease (COPD) [4, 5], interstitial lung disease [6], bronchiectasis [7], heart failure [8] and pneumonia [9–11]. Positive findings during auscultation influence clinical decisions such as the rate of antibiotic prescriptions [12, 13] and referrals to specialist care [14].

Presence of ALS alone, however, only show moderate sensitivities and specificities, limiting their diagnostic utility [15–17]. This modest accuracy is mainly related to the fact that both wheezes and crackles can also be present in apparently healthy adults [10, 18–20]. To determine the real usefulness of ALS it is crucial to define first their behavior, presence and characteristics, in apparently healthy people. Most studies to date, however, have investigated how ALS relate to specific diagnostic categories without considering their distribution across the whole spectrum from health to disease. Moreover, the few existent studies investigating ALS in apparently healthy people used small samples [19], failing to be representative of the general population. The prevalence of wheezes and crackles in a general population has never been reported. [21].

With this study, we aimed to estimate the prevalence of wheezes and crackles in a large general adult population. We also explored to which degree ALS are associated with self-reported disease, smoking status and clinical measures of lung function.

## Methods

### Design and participants

The Tromsø Study is an epidemiological survey that started in 1976 with the main goal to determine the reasons for the high cardiovascular mortality in the municipality of Tromsø, Norway. The study has been periodically repeated with the last survey (7th) taking place in 2015–16.Details of the Tromsø Study can be consulted elsewhere [22, 23].

In this cross-sectional study, our sample consisted of randomly selected participants attending the second visit of the seventh survey of the Tromsø study (Tromsø 7), between May 2015 and October 2016. All Tromsø residents 40 years and older (*n* = 32,591) received a postal invitation to participate in the first visit of Tromsø 7. A random sample was selected for the second visit including 20% of those aged 40–59 years and 60% of those aged 60–84 years, and those attending the first visit were invited. In addition, individuals who had participated in previous surveys of the study were invited to obtain repeated measurements. The mean time between the visits was of 52 days (± 32). All study participants provided written consent. The Regional Committee for Medical and Health Research Ethics in North Norway approved the study.

### Questionnaires and examinations

In the first visit, the participants filled a questionnaire that included questions on medical conditions such as arterial hypertension, heart failure, atrial fibrillation, COPD, asthma, among others. For each condition, the participants were asked to specify if it was a current diagnosis, if they had that diagnosis at some point in the past or if they never had that diagnosis. They also responded questions about smoking habits. The full questionnaires employed at the Tromsø Study can be consulted in English elsewhere [22].

At the second visit, the participants answered the modified Medical Research Council questionnaire (mMRC) on dyspnea [24]. Dyspnea was further characterized using the question: “How is your breathing today compared to normal?”. To better characterize the respiratory status, participants were also asked if they had respiratory infection in the previous week (“Have you had symptoms of common cold, bronchitis or other airway infection the last 7 days?”).

Spirometry was performed using SensorMedics Vmax 20c Encore (VIASYS Healthcare Respiratory Technologies, Yorba Linda, CA, USA). Calibration was done daily. We followed the standards of the American Thoracic Society (ATS)/ European Respiratory Society (ERS) [25]. Tests with FEV1 < 0.3 l and with expiration lasting for less than 3 s were regarded invalid. We did not perform post-bronchodilator measurements. We used the Global Lung Function Initiative (GLI 2012) as a reference [26]. We registered arterial oxygen saturation (Sp0_2_) with a pulse oximeter Onyx II model 9550 (Nonin Medical, Inc., Plymouth, MN, USA) after resting 15 min. The highest value after three measurements was registered. We accepted only SpO_2_ ≥ 80% due to uncertain validity of lower values (*n* = 1). At the end of this second visit we recorded lung sounds.

### Recording of the lung sounds

We used a microphone MKE 2-EW with a wireless system EW 112-P G3-G (Sennheiser electronic GmbH, Wedemark, Germany), placed in the tube of a Littmann Classic II stethoscope (3 M, Maplewood MN, USA) at 10 cm from the headpiece. The signal went to an external sound card (Scarlett 2i2, Focusrite Audio Engineering Ltd., High Wycombe UK) which connected to a computer’s audio input. The computer used custom developed software to label the sounds (participant ID, recording site) and allowed us to start the recording with a wireless control (R700, Logitech Europe S.A., Lausanne Switzerland).

**Fig. 1:**
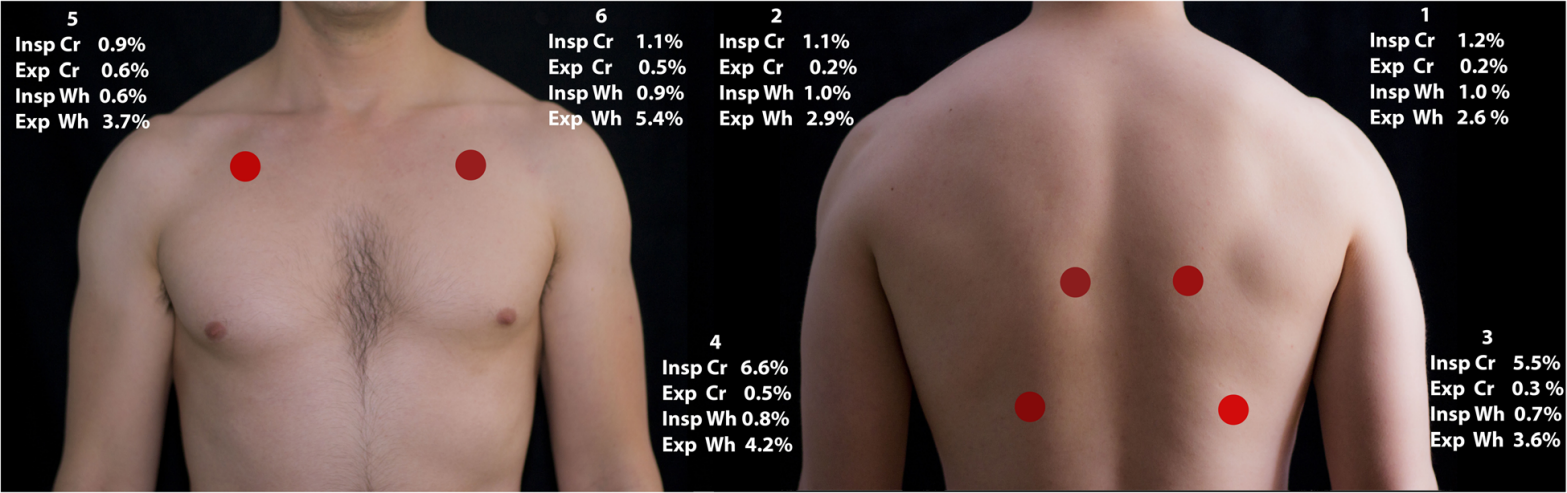
Recording sites and prevalence of findings. (1 and 2) Between the spine and the medial border of the scapula at the level of T4–T5; (3 and 4) at the middle point between the spine and the mid-axillary line at the level of T9–T10; (5 and 6) at the intersection of the mid-clavicular line and second intercostal space. Cr = crackles, Wh = wheezes, Insp = inspiratory, Exp = expiratory

We recorded in a quiet room with the participants sitting and the thorax exposed. They were asked to breathe deeper than normal with an open mouth. We started the recordings on inspiration and recorded for 15 s. We performed the same procedure subsequently at six different locations (Fig. 1). The quality of the recordings was monitored using a wireless headset (SDR 160, Sennheiser electronic GmbH, Wedemark, Germany). If the health professional deemed the quality to be unsatisfactory, a second attempt was performed.

We obtained audio files in “.wav” format at a sample rate of 44,100 Hz and 16-bit depth in a single (monophonic) channel. We did not implement audio filters or other digital pre or post-processing techniques.

### Classification of the recordings

The classification process consisted of three steps.

At the first step, two observers independently listened to all the recordings with a headset and simultaneously viewed the sound spectrograms using Adobe Audition 5.0 (Adobe Systems, San Jose, CA, USA). J.C.A. was observer 1 and either R.E., A.D. or C.J. were observer 2. They evaluated if the recording contained wheezes (including rhonchi), crackles or other ALS and whether these were heard in inspiration or expiration. They entered their findings in an electronic form (Access, Microsoft Corporation, Redmond WA, USA) and registered if artefactual noise made the classification difficult. The observers could listen to the recordings with freedom to stop or repeat parts or the whole recording if necessary. They were blinded to any information about the participant. Agreement and kappa statistics were calculated accounting for the clustered structure of the data using the R package “magree”. [27].

At the second step, all disagreements were evaluated with the two initial observers and a third experienced observer (H.M.). The three observers listened to the sounds and solved disagreements through consensus. If consensus was difficult to reach at this point, the sounds were submitted for classification at the third step.

At the third step, all recordings classified as containing ALS were re-classified by two pairs of observers consisting of one junior (J.C.A. and C.J.) and senior (H.P. and H.M.) lung sound researcher each. These observers had the possibility to mark the findings as “certain”, “possible” or “absent”. A finding was changed into absent when wheezes or crackles were marked as “absent” or “possible” by both observers. Findings classified as “present” by one observer and “absent” by the other were discussed in a face-to-face meeting with all the four observers. Agreement between at least three out of four observers was required to classify an ALS as “present”. At the same session, difficult sounds from step two and sounds categorized as “other sounds” were classified.

All observers performed an audiometry at the time of involvement in the project. All observers had normal hearing.

### Statistical methods

We calculated age-standardized prevalence of wheezes and crackles in men and women using the population distribution from the municipality of Tromsø per January 2018 [28]. The ALS were divided into three categories: wheezes and no crackles, crackles and no wheezes, and both wheezes and crackles, irrespective of respiratory phase. We calculated prevalence by participant characteristics and used linear models to explore statistically significant differences among the groups. Tukey’s procedure was used to account for multiple testing. The continuous variables were dichotomized with cutoff values for age ≥ 65 years, for oxygen saturation ≤ 95% [29], mMRC score ≥ 2 [30], Body Mass Index (BMI) ≥30 (obesity threshold) and FEV_1_ below the lower limit of normal (LLN), according to the Global Lung Initiative reference [26].

We used univariable logistic regression to study wheezes and crackles in relation to the variables of interest. In this analysis, wheezes were counted as present also when accompanied by crackles and vice versa. The following outcome variables were considered separately: (1) any wheeze, (2) wheezes only during the inspiratory phase, (3) wheezes during the expiratory phase and (4) wheezes during the expiratory phase at two or more recording sites. For crackles, the outcomes were (1) any crackle, (2) inspiratory crackles, (3) inspiratory crackles at two or more locations, (4) only expiratory crackles. The categorical variables of FEV_1_ < LLN and SpO_2_ ≤ 95% were substituted by continuous data (FEV_1_ Z-score and SpO_2_%) to avoid loss of information. We divided age per decades and kept it as a continuous variable. The variables of self-reported disease were dichotomized as present (which included both present or past diagnosis) and absent (never diagnosed).

All statistically significant variables for each outcome in the univariable analyses were entered into multivariable logistic regression models. We performed a backward elimination procedure with a threshold of *p* < .05 to obtain the best fitting models for each outcome. We plotted Receiver Operator Characteristics (ROC) curves for all the final models and calculated the area under the curve (AUC) with the r package “pROC” [31]. Multicollinearity in the final models was assessed using variance inflation factor with the statistical package “car” [32]. We used R statistical computing version 3.2.1 package to perform all the calculations [33]. Results were considered significant at 5% level.

## Results

### Participants

Tromsø 7 had an attendance of 21,083 (65%) in the first visit [22]. Of these, 9253 had been selected in advance to be invited to the second visit, and 90% (*n* = 8346) took part. Limited by absences of the staff, we recorded lung sounds in 6035 (72.3%). Restricted by human resources and time constraints, only 4033 participants were included in the classification procedure. Our final number of participants represents 19.1% of the participation in Tromsø 7 and 48.3% of those attending the second visit. A comparison of the main characteristics between all the participants of Tromsø 7 and the final study sample and the flow diagram of the participants included in our analyses are available online. (Additional file 1: Table S1, Additional file 2: Figure S1).

### General characteristics of the groups

The mean age of all 4033 participants was 63.5 years, and 2159 (53.5%) were female. (Table 1). There were 477 (11.0%) and 2372 (47%) current and previous smokers, respectively. We found an FEV_1_ < LLN in 286 (7.1%) participants and 182 (4.5%) had oxygen saturation ≤ 95% (Table 1). We observed that women had lower proportion of myocardial infarction, heart failure and past smokers, but they presented a higher proportion of self-reported asthma, dyspnea (mMRC) and oxygen saturation ≤ 95%.

**Table 1:** Characteristics of the study population

	Male (*n* = 1874) n (%)	Female (*n* = 2159) n (%)	Missing (*n* = 4033) n (%)
Age	63.7 (±10.5)	63.4 (±10.7)	
< 65 years	908 (48.5%)	1071 (49.6%)	
≥ 65 years	966 (51.5%)	1088 (50.4%)	
Body-mass index			12 (0.3%)
< 30	1425 (76.0%)	1683 (78.0%)	
≥ 30	445 (23.7%)	468 (21.7%)	
Smoking status			59 (1.5%)
Never smoker	686 (36.6%)	916 (42.4%)***	
Current smoker	208 (11.1%)	269 (12.5%)	
Previous smoker	954 (50.9%)	941 (43.6%)***	
Self-reported disease			
Hypertension	473 (25.2%)	557 (25.8%)	119 (3.0%)
Myocardial Infarction	141 (7.5%)	57 (2.6%)***	171 (4.2%)
Heart failure	33 (1.8%)	16 (0.7%)**	175 (4.3%)
Atrial Fibrillation	92 (4.9%)	80 (3.7%)	178 (4.4%)
COPD	74 (3.9%)	87 (4.0%)	157 (3.9%)
Asthma	128 (6.8%)	196 (9.1%)**	254 (6.3%)
Rheumatoid arthritis	83 (4.4%)	117 (5.4%)	227 (5.6%)
Airways infection last week §	278 (14.8%)	303 (14.0%)	165 (4.1%)
Dyspnea			
mMRC			165 (4.1%)
mMRC 0	1323 (70.6%)	1368 (63.4%)***	
mMRC 1	412 (22.0%)	575 (26.6%)***	
mMRC 2–4	70 (3.7%)	120 (5.6%)**	
Breathing worse than usual §	210 (11.2%)	242 (11.2%)	156 (3.9%)
Oxygen saturation, SpO_2_			161 (4.0%)
≤ 95%	110 (5.9%)	72 (3.3%)***	
Spirometry			
FEV_1_ < LLN †	150 (8.0%)	136 (6.3%)	234 (5.8%)

Abbreviations: *mMRC* = Modified Medical Research Council questionnaire, *FEV*_*1*_ = Forced Expiratory Volume in one second, *LLN* = Lower Limit of Normal

§On examination day

***p value <.001, **p value <.01, *p value <.05 as compared to male by X^2^ test

### Classification agreement

We included 24,198 (4033 × 6 recording sites) recordings for classification. At the first step the observers agreed on inspiratory wheezes in 98.7% of the recordings (kappa (k) = 0.43; 95%CI 0.37–0.49), on expiratory wheezes 96.2% (k = 0.56; 0.53–0.59), on inspiratory crackles in 96.5% (k = 0.46; 0.42–0.49), and on expiratory crackles in 98.5% (k = 0.20; 0.15–0.25). Examples of the recordings can be consulted online (Additional file 3: Figure S3).

At the second step, 1257 recordings were marked as containing wheezes and 894 containing crackles. At the third step we discarded wheezes in 224 of these recordings and crackles in 174.

The presented prevalence of ALS are based on six recordings in 3771 (93.5%) participants. However, in 262 (6.5%) of the participants included in the analysis there was noise in one or more recordings. Five recording sites were considered in 223 (5.5%) participants and four or less recording sites in 39 (1%) participants.

### Prevalence of wheezes and crackles

We found 28% (*n* = 1131) of individuals with ALS at least at one recording site. Of these, 599 (14.9%) had only wheezes, 402 (10.0%) had only crackles and 130 (3.2%) had both wheezes and crackles (Table 2). Expiratory wheezes and inspiratory crackles were the most common findings (Fig. 1). Of the 729 participants with wheezes, 534 (73.3%) had wheezes at one location, 132 (18.1%) at two locations, 63 (8.6%) at three or more locations. Of the 532 participants with crackles, 381 (71.6%) had crackles at one recording site, 127 (23.9%) at two recording sites, 24 (4.5%) at three or more recording sites. Inspiratory crackles were more frequent at the bases (Fig. 1).

**Table 2:** Frequency of wheezes, crackles and both by characteristics of the study population

	Normal	Wheezes, no crackles	Crackles, no wheezes	Both crackles and wheezes
	n (%)	n (%)	n (%)	n (%)
All (*n* = 4033)	2902 (72.0%)	599 (14.9%)	402 (10.0%)	130 (3.2%)
Age				
< 65 years	1539 (77.8%)	277 (14.0%)**	123 (6.2%)***	40 (2.0%)***
≥ 65 years	1363 (66.4%)	322 (15.7%)	279 (13.6%)	90 (4.4%)
Gender				
Male	1389 (74.1%)	190 (13.4%)	251 (10.1%)	44 (2.3%)
Female	1513 (70.1%)	348 (16.1%) *	212 (9.8%)	86 (4.0%)**
Body-mass index				
< 30	2232 (71.8%)	483 (15.5%)*	292 (9.4%)	101 (3.2%)
≥ 30	663 (72.6%)	114 (12.5%)	107 (11.7%)	29 (3.2%)
Smoking status				
Never smoker	1209 (75.5%)	227 (14.2%)	133 (8.3%)**	33 (2.1%)**
Current smoker	299 (62.7%)	89 (18.7%) **	64 (13.4%) **	25 (5.2%) **
Previous smoker	1349 (71.2%)	278 (14.7%)	198 (10.4%)	70 (3.7%)
Self-reported disease				
Healthy †	1177 (76.8%)	218 (14.0%)	120 (7.0%)***	38 (2.4%)*
Hypertension	722 (70.1%)	151 (14.7%)	120 (11.7%)	37 (3.6%)
Myocardial Infarction	123 (62.1%)	35 (17.7%)	30 (15.2%) *	10 (5.1%)
Heart failure	32 (65.3%)	11 (22.4%)	5 (10.2%)	1 (2.0%)
Atrial Fibrillation	114 (66.3%)	29 (16.9%)	25 (14.5%)	4 (2.3%)
COPD	95 (59.0%)	27 (16.8%)	30 (18.6%) ***	9 (5.6%)
Asthma	209 (64.5%)	55 (17.0%)	38 (11.7%)	22 (6.8%) **
Rheumatoid arthritis	129 (64.5%)	29 (14.5%)	30 (15.0%) *	12 (6.0%)
Airways infection last week §	427 (73.5%)	89 (15.3%)	44 (7.6%)*	21 (3.6%)
Dyspnea				
mMRC				
mMRC 0	1996 (74.2%)	388 (14.4%)	239 (8.9%)**	68 (3.7%)**
mMRC 1	688 (69.7%)	148 (15.0%)	111 (11.2%)	40 (4.1%)
mMRC 2–4	109 (57.4%)	30 (15.8%)	36 (18.9%) ***	15 (7.9%) ***
Breathing worse than usual §	321 (71.0%)	70 (15.5%)	47 (10.4%)	14 (3.1%)
Oxygen saturation SpO_2_				
≤ 95%	106 (58.2%)	25 (13.7%)	35 (19.1%)***	16 (8.8%)***
Spirometry				
FEV_1_ < LLN ‡	175 (61.2%)	52 (18.2%)	40 (14.0%)*	19 (6.6%)***

Abbreviations: *mMRC* = Modified Medical Research Council questionnaire, *FEV*_*1*_ = Forced Expiratory Volume in one second, *LLN* = Lower limit of Normal

Plus-minus values are means + − SD

Percentages (%) represent the distribution of each variable between the different groups

***p value <.001, **p value <.01, *p value <.05 as compared to normal

† Not current smokers who stated not to have any of the diseases considered for this analysis

‡ Calculated from Global Lung Function Initiative reference (GLI)

§ On examination day

**Fig. 2:**
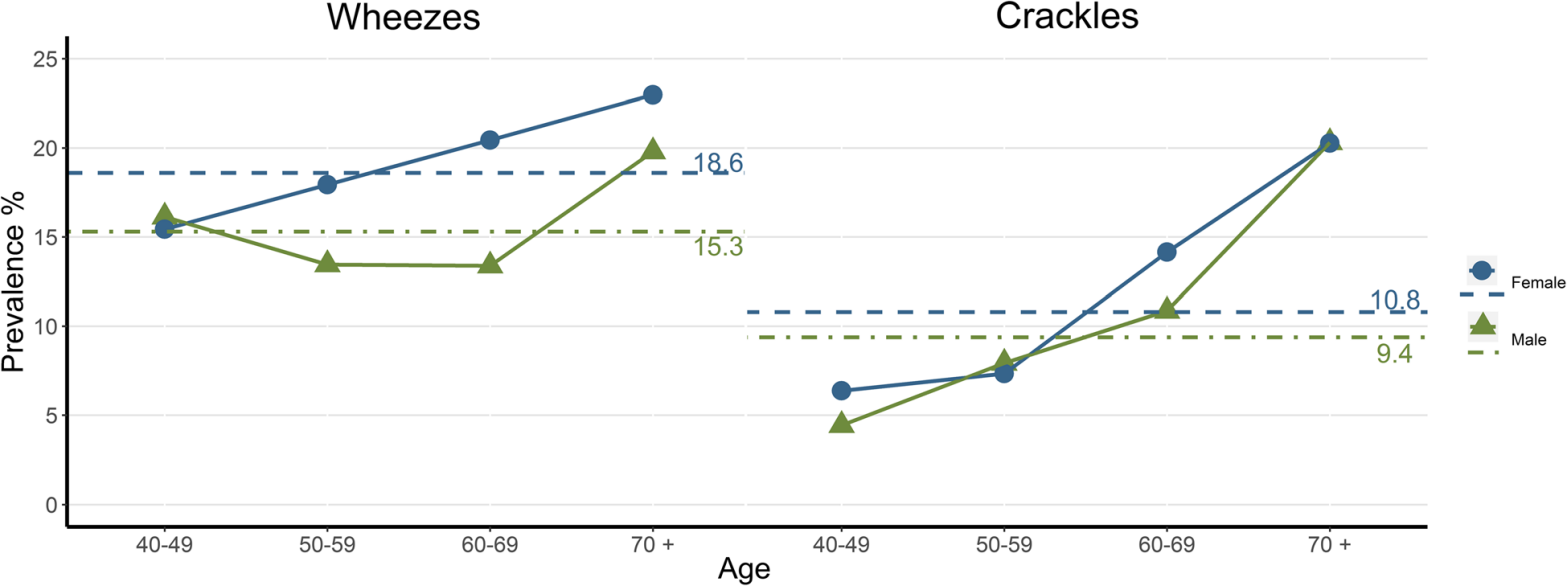
Prevalence of wheezes and crackles by age (in years). Dotted lines represent standardized prevalence rates

The age-standardized prevalence of wheezes was 18.6% for women and 15.3% for men and of crackles, 10.8 and 9.4%, respectively. The prevalence of ALS increased significantly with age in both men and women (*p* < .001). This was particularly the case for crackles (Fig. 2). Pleural rub and bronchial breathing were rarely noticed, each in only two participants.

Wheezes or crackles were found in more than 40% of participants with the following characteristics: self-reported COPD, mMRC≥2, FEV_1_ < LLN and SpO_2_ ≤ 95% (Table 2). These characteristics were also associated with the highest prevalence of having both wheezes and crackles, 6.6–8.8% (Table 2).

### Predictors of wheezes

In the univariable analysis, we found that wheezes were associated with age (10 years increase), female gender, self-reported asthma, current smoking, mMRC≥2, and a reduction in FEV_1_ Z-score, (Table 3). The associations with mMRC≥2, current smoking and FEV_1_ Z-score were stronger for inspiratory than for expiratory wheezes. In the multivariable analysis age, female gender, self-reported asthma, and current smoking predicted the occurrence of expiratory wheezes (Table 4). FEV_1_-Z score was a significant predictor for the occurrence of inspiratory wheezes. The AUC for all the multivariable models were similar (0.59–0.60, Table 4). Multicollinearity was not problematic since the maximum variance inflation factor was < 1.07.

**Table 3:** Odds ratio for the occurrence of crackles and wheezes in univariable regression models

	Wheezes OR (95% CI)				Crackles OR (95% CI)			
	Any Wheezes (*n* = 729)	InspiratoryWheezes (*n* = 151)	Expiratory wheezes (*n* = 649)	Expiratory wheezes at 2 or more locations (*n* = 167)	Any crackles (*n* = 532)	Inspiratory crackles (*n* = 495)	Expiratory crackles (*n* = 79)	Inspiratory crackles at 2 or more locations (*n* = 135)
Age (10 years)	1.2*** (1.1–1.2)	1.1 (0.9–1.2)	1.2*** (1.1–1.3)	1.2** (1.1–1.4)	1.7*** (1.6–1.9)	1.8*** (1.6–2.0)	1.4** (1.1–1.8)	2.2*** (1.8–2.8)
Female	1.3*** (1.1–1.6)	1.2 (0.8–1.6)	1.4*** (1.2–1.7)	1.5* (1.1–2.1)	1.1 (0.9–1.3)	1.1 (0.9–1.4)	1.7* (1.1–2.7)	1.1 (0.8–1.5)
BMI > 30	0.8* (0.7–1.0)	0.8 (0.5–1.1)	0.8 (0.7–1.0)	0.9 (0.6–1.3)	1.2 (1.0–1.5)	1.2* (1.0–1.5)	0.6 (0.3–1.1)	1.5* (1.0–2.2)
Hypertension	1.0 (0.8–1.2)	1.2 (0.8–1.7)	1.0 (0.8–1.2)	1.2 (0.8–1.6)	1.3* (1.0–1.6)	1.3* (1.1–1.6)	0.9 (0.5–1.5)	1.2 (0.8–1.7)
Myocardial Infarction	1.3 (0.9–1.9)	1.5 (0.8–2.7)	1.3 (0.9–1.9)	1.4 (0.7–2.4)	1.7** (1.2–2.5)	1.7** (1.2–2.5)	1.0 (0.3–2.5)	1.8 (0.9–3.3)
Heart Failure	1.5 (0.7–2.8)	1.7 (0.4–4.7)	1.3 (0.6–2.6)	0.5 (0.0–2.2)	0.9 (0.4–2.0)	1.0 (0.4–2.2)	< 0.1 (< 0.1 - > 100)	0.6 (0.0–2.9)
Atrial fibrillation	1.1 (0.7–1.6)	1.3 (0.6–2.4)	0.9 (0.6–1.4)	1.1 (0.5–2.2)	1.4 (0.9–2.0)	1.4 (0.9–2.2)	0.3 (< 0.1–1.2)	1.7 (0.8–3.2)
COPD	1.3 (0.9–1.9)	1.5 (0.7–2.9)	1.3 (0.8–1.9)	1.6 (0.8–2.9)	2.3*** (1.5–3.2)	2.4*** (1.6–3.4)	1.6 (0.6–3.7)	5.6*** (3.3–8.9)
Asthma	1.5** (1.1–1.9)	1.5 (0.8–2.4)	1.4* (1.0–1.8)	1.8* (1.1–2.8)	1.6** (1.1–2.1)	1.4* (1.0–2.0)	1.8 (0.9–3.4)	1.7* (1.0–2.8)
Rheumatoid Arthritis	1.2 (0.8–1.7)	1.2 (0.6–2.2)	1.1 (0.7–1.5)	0.7 (0.3–1.5)	1.8** (1.3–2.6)	1.9** (1.3–2.7)	1.6 (0.6–3.5)	1.6 (0.8–3.6)
Current smoker	1.6*** (1.3–2.1)	2.1** (1.3–3.3)	1.6** (1.2–1.8)	1.7* (1.0–2.6)	2.0*** (1.5–2.6)	2.0*** (1.5–2.7)	1.3 (0.6–2.5)	2.5** (1.5–2.6)
Previous smoker	1.2 (1.0–1.4)	1.1 (0.8–1.6)	1.1 (1.0–1.2)	1.2 (0.8–1.6)	1.4** (1.2–1.8)	1.5*** (1.2–1.9)	0.9 (0.6–1.5)	2.0** (1.3–2.1)
mMRC ≥ 2	1.5* (1.0–2.0)	2.0* (1.1–3.5)	1.4 (0.9–1.9)	1.5 (0.7–2.6)	2.6*** (1.8–3.6)	2.7*** (1.9–3.7)	1.7 (0.7–3.7)	3.4*** (1.9–5.5)
Breathing worse than usual	1.1 (0.8–1.4)	1.4 (0.9–2.2)	1.0 (0.7–1.3)	1.0 (0.6–1.5)	1.0 (0.8–1.4)	1.1 (0.8–1.4)	0.8 (0.3–1.6)	1.7* (1.0–2.6)
Symptoms of airways infection last week	1.1 (0.9–1.4)	1.4 (0.9–2.1)	1.0 (0.8–1.3)	1.4 (0.9–2.0)	0.8 (0.6–1.1)	0.9 (0.6–1.1)	0.8 (0.4–1.5)	1.2 (0.8–1.9)
Oxygen Saturation SpO_2_ (1%)	1.0 (0.9–1.0)	0.9 (0.8–1.0)	1.0 (0.9–1.0)	0.9 (0.9–1.1)	0.8*** (0.7–0.9)	0.8*** (0.7–0.8)	1.0 (0.9–1.3)	0.7*** (0.6–0.8)
FEV_1_ Z-score (1 unit)	0.9** (0.8–1.0)	0.7*** (0.6–0.8)	0.9* (0.8–1.0)	0.8* (0.7–1.0)	0.8*** (0.7–0.9)	0.8*** (0.7–0.9)	0.9 (0.7–1.1)	0.6*** (0.5–0.7)

Confidence intervals shown in brackets. mMRC = Modified Medical Research council questionnaire. FEV1 = Forced expiratory volume in 1 s. LLN = Lower Limit of Normal

***p value < .001, **p value < .01, *p value < .*05*

**Table 4:** Odds ratio for the occurrence of crackles and wheezes in multivariable regression models

	Inspiratory wheezes (*n* = 130)	Expiratory wheezes (*n* = 587)	Expiratory wheezes 2+ (*n* = 151)	Inspiratory crackles (*n* = 445)	Expiratory crackles (*n* = 70)	Inspiratory crackles 2+ (*n* = 118)
Age (×0.1)	–	1.2*** (1.1–1.3)	1.2** (1.1–1.5)	1.8*** (1.6–2.0)	1.5** (1.2–1.9)	2.2*** (1.8–2.9)
Female Gender	–	1.5*** (1.2–1.8)	1.5* (1.1–2.1)	–	–	–
COPD	–	–	–	–	–	2.3** (1.3–4.1)
Asthma	–	1.4* (1.0–1.8)	1.9** (1.1–3.0)	–	–	–
Rheumatoid Arthritis	–	–	–	1.6* (1.1–2.3)	–	–
Current smoker	–	1.7*** (1.3–2.2)	–	1.9*** (1.4–2.7)	–	–
Previous smoker	–	1.1 (0.9–1.3)	–	1.3* (1.0–1.6)	–	–
mMRC ≥ 2	–	–	–	1.8** (1.2–2.6)	–	–
Oxygen saturation SpO_2_ (1%)	–	–	–	0.9** (0.8–1.0)	–	0.7*** (0.6–0.8)
FEV_1_ Z-score (1 unit)	0.7*** (0.6–0.8)	–	–	0.9** (0.8–1.0)	–	0.7*** (0.6–0.8)
AUC	.59 (0.54–0.64)	.59 (0.56–0.62)	.60 (0.55–0.64)	.69 (0.67–0.72)	.62 (0.56–0.69)	.79 (0.75–0.84)

Confidence intervals shown in brackets. 2+ = presence of the adventitious sounds in more than two locations. *mMRC* = Modified Medical Research council questionnaire. *FEV1* = Forced expiratory volume in 1 s, *AUC* = Area under the curve

***p value < .001, **p value < .01, *p value <.05

### Predictors of crackles

**Fig. 3:**
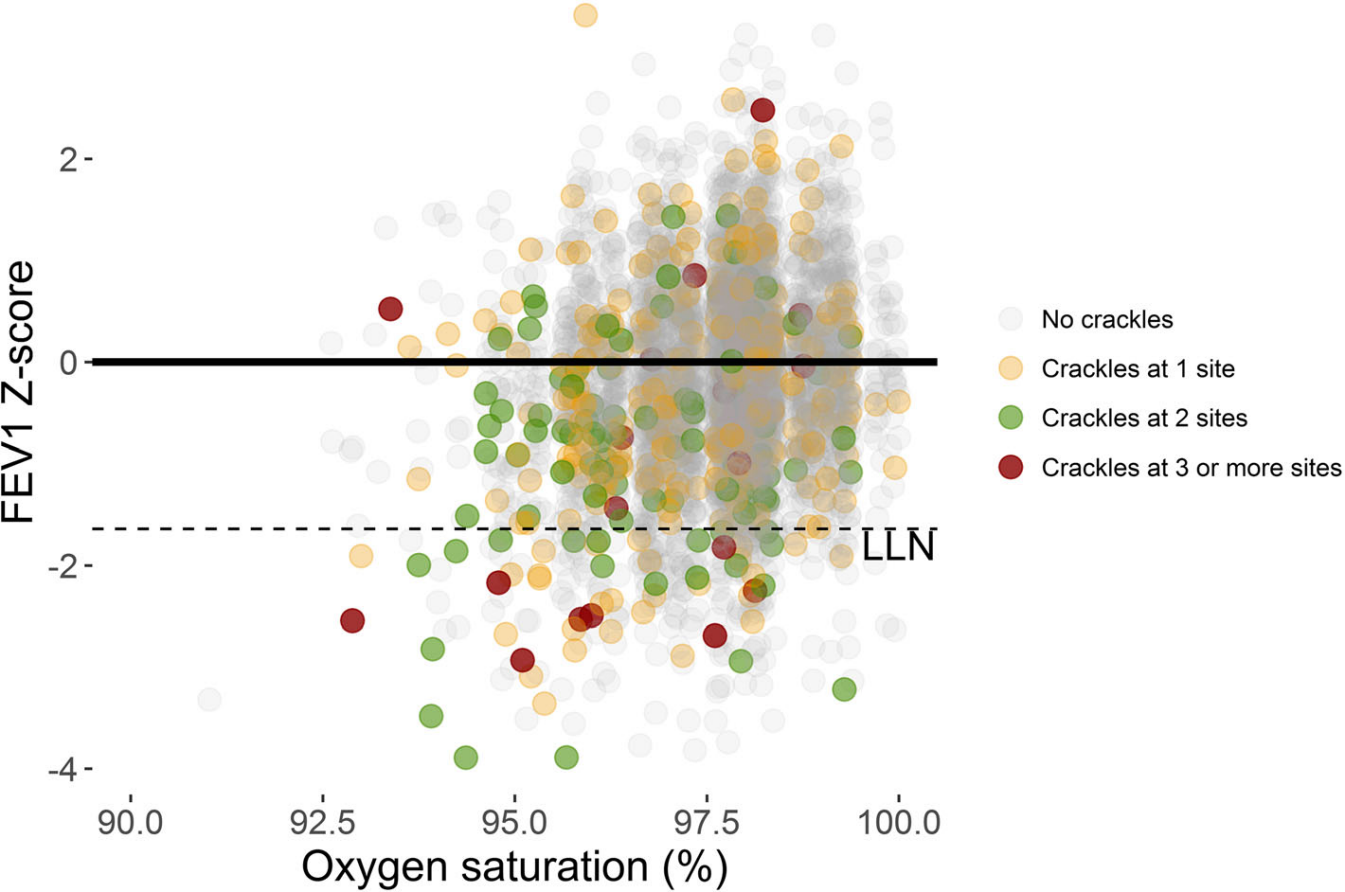
Occurrence of Inspiratory crackles by Z-score FEV1 (reference GLI 2012) and SpO2. LLN = Lower Limit of Normal

The explanatory variables were stronger predictors of crackles than of wheezes (Table 3). Age and gender were the only variables associated with expiratory crackles. For inspiratory crackles, the effect of age, self-reported COPD, asthma, current and previous smoking, mMRC ≥2, oxygen saturation and FEV_1_ Z-score was stronger when inspiratory crackles were found at two or more recording sites than for inspiratory crackles at one site only. Similarly, in the multivariable analysis the strongest associations were found in the model with inspiratory crackles heard at two or more sites as outcome. This was the model with the highest area under the curve (AUC = 0.79). Inspiratory crackles appeared more often and at more locations in individuals with a negative FEV_1_ Z-score and low oxygen saturation (Fig. 3). Multicollinearity was not problematic since the maximum variance inflation factor was < 1.01.

### Predictors of wheezes and crackles in the same subject

In the multivariable analysis with both wheezes and crackles as outcome, age, female gender and FEV_1_ Z-score were the significant predictors (data not shown). The AUC of the model was 0.7.

The variables “respiratory infection previous week” and “more short of breath than usual” predicted neither wheezes nor crackles.

## Discussion

Wheezes and crackles were common findings. Any of these sounds were found in almost one third of our sample. Wheezes and crackles were associated with increasing age. The sounds were not always related to clinically diagnosed disease, but their prevalence increased in the presence of decreased lung function or chronic shortness of breath.

We are not aware of any comparable study carried out in a general population. An investigation with 700 participants conducted by Murphy et al., found wheezes and rhonchi in 4 and 4%, respectively, in a subgroup of 334 apparently healthy adults. Wheezes were heard in 59% of patients with asthma. For crackles, the prevalence was 21% in the apparently healthy group and 71% in patients with COPD. [10] These prevalences were higher than what we observed, except for wheezes in the apparently healthy group. In their study, the age of the participants was not taken into account. They included more recording sites than in ours and used a computerized classification of the sounds. Different sensitivities of the classification methods may partly explain the discordance in prevalence. Crackles detected by a computer algorithm may be inaudible with a stethoscope since crackles may be masked by normal sounds. [34].

In most participants with ALS in our study, these were heard at only one of the six recording sites. The number of sites with positive findings had an impact on the associations. The model with inspiratory crackles at two or more locations as outcome performed better than the model predicting any crackles, reaching an AUC of 0.79. No similar effect of increasing number of sites was found regarding wheezes.

The importance of age was described by Kataoka et al., who observed a rising prevalence of crackles from 11% in cardiovascular asymptomatic adults 40–65 years to 70% in participants 80–95 years old. [35] Murphy et al. found an association with age among asbestos exposed workers. [36] Age relates to a reduction of supporting tissue around the airways causing a premature closure of the airways. [37]. The influence of lung and heart disease associated with ageing might have contributed to the strong association between crackles and age in our study, an influence beyond what indicated by self-reported diseases, spirometry and pulse oximetry.

Self-reported asthma was associated with wheezes, which was in line with previous studies. [3] Hypertension, self-reported asthma, myocardial infarction, self-reported COPD, and RA were associated with crackles in the univariable analysis, but only the latter two remained statistically significant in the multivariable models. The association of crackles with RA could be explained by the presence of parenchymal lung abnormalities in patients with this diagnosis. [38] However, we did not have an independent confirmation of the diagnosis. Self-reported heart failure was not associated with crackles probably due to underdiagnosed heart failure [39]. Interestingly, symptoms suggestive of airway infection the week before the examination was not an independent predictor of crackles or wheezes. In a European study from primary care of 2810 adults with acute cough, crackles were registered in 31% of patients in the pneumonia group. [40] Since the prevalence of pneumonia in this study was only 5%, and probably far less in our study, it is likely that crackles represented chronic rather than acute changes in the lungs in most cases.

Women had a higher prevalence of wheezes than men. Considering subcategories of wheezes, this observation was valid for expiratory but not for inspiratory wheezes. The same gender disparity has been reported in epidemiological studies on self-reported wheeze [41, 42]. Although wheezing is more common in male newborns and infants, this gender difference seems to change sometime during adolescence when females start to show a higher risk of wheezing. [43] Our findings indicate that this may persist into later adulthood.

Oxygen saturation was significantly associated with the presence of inspiratory crackles. Crackles are related to the sudden opening of closed airways or to air movement through obstructed airways. [44] These conditions may impair ventilation/perfusion matching, the most common cause of hypoxemia, which could explain the relationship in our study. [37].

### Strengths and limitations

To our knowledge, this is the largest sample characterizing the occurrence of wheezes and crackles to date. Tromsø 7 had a high response rate (65%). The study has a high external validity for the Norwegian population. [23] Nevertheless, our results might not be valid in other populations, for instance in those with poorer health. Unhealthy people may be underrepresented since some might have chosen to refrain from participation or were not able to attend and complete the survey.

The questionnaires employed at Tromsø 7 did not ask about the presence of interstitial lung disease and bronchiectasis. Both conditions have an increased prevalence with age [45, 46] and are associated with the presence of crackles [6, 7]. It is possible that participants with these conditions were categorized as apparently healthy and this constitutes a limitation of our study.

All the selection processes were randomized and took place prior to the classification of the recordings without having any knowledge of the health status of the participants. When randomizing for the second visit a higher representation was chosen among those aged 60 years or more, and the subjects invited due to participation in previous surveys of the Tromsø study were usually 60 years or older. In terms of prevalence, we have taken care of this selection bias by age standardization, but some influence on associations with self-reported diseases and lung function cannot be excluded.

The inter-observer agreement at the first step of our classification compares to that found among general practitioners. [47] The repeated independent classifications have without doubt increased the reliability. [48] A lack of reliability could have influenced our results by diluting the strength of the estimates. At the third step of the classification, we chose to discard as positive findings the recordings marked as “possible” by two observers. In a sensitivity analysis, they were classified as “present” but the results were similar, and our conclusions unchanged.

## Conclusion

Our findings support a cautious attitude when using ALS to diagnose lung disease in elderly patients. The presence of wheezes or crackles in one lung location did not strongly predicted the outcomes analyzed. Nonetheless, it is possible that these solitary findings are a manifestation of lung senescence and/or represent subclinical disease in apparently healthy subjects. However, when inspiratory crackles at two or more locations or both wheezes and crackles are heard, risk of decreased lung function increases considerably. Such findings, particularly when unexpected in a patient, should lead to further investigation regarding possible heart or lung disease.

## Additional files

Additional file 1: **Table S1.** Comparison between all the participants attending the 7th Survey of the Tromsø Study and the final sample included in this article. (DOCX 12 kb)

Additional file 2: **Figure S1.** Flow diagram of the participants included in our analyses. (JPG 292 kb)

Additional file 3: **Figure S2.** Spirogram and clinical information of four participants with presence of adventitious lung sounds in the recordings. (PPTX 2605 kb)

## Data Availability

The data that support the findings of this study are available from The Tromsø Study but restrictions apply to the availability of these data, which were used under license for the current study, and so are not publicly available. Information and guidelines to apply for access to data from The Tromsø study are available at https://en.uit.no/prosjekter/prosjekt?p_document_id=71247.

## Abbreviations

ALS: Adventitious Lung Sounds
AUC: Area Under the Curve
COPD: Chronic Obstructive Pulmonary Disease
FEV_1_: Forced Expiratory Volume in 1 s
GLI: Global Lung Initiative
LLN: Lower Limit of Normal
mMRC: Modified Medical Research Council dyspnea scale
OR: Odds Ratio
RA: Rheumatoid Arthritis
SpO_2_: Arterial oxygen saturation
