# Supplementary material for: Prevalence and clinical associations of wheezes and crackles in the general population: the Tromsø study
Source: BMC Pulm Med. 2019 Sep 11;19:173. doi: 10.1186/s12890-019-0928-1 (PMC6739986; doi:10.1186/s12890-019-0928-1)
Supplement: Supplementary file 1 — Table S1. Comparison between all the participants attending the 7th Survey of the Tromsø Study and the final sample included in this article. (DOCX 12 kb) [file 12890_2019_928_MOESM1_ESM.docx]

|  | **All participants Tromsø 7**  **(n=21 083)** | **Final sample**  **(n= 4 033)** |
| --- | --- | --- |
| **Age** | 57.3 (+/-11.4) | 63.55 (+/-10.6) |
| **Female sex** | 52.5 % | 53.5 % |
| **Weight** | 79.9 (+/- 16) | 78.6 (+/- 15.4) |
| **Smoke never** | 41.8 % | 39.7 % |
| **Current smoker** | 13.9 % | 11.8 % |
| **Past smoker** | 44.3 % | 47 % |
| **Hypertension** | 28.4 % | 25.5% |
| **Myocardial Infarction** | 3.7 % | 4.9 % |
| **Atrial Fibrilation** | 6.7 % | 4.3 % |
| **Heart Failure** | 1.9 % | 1.2 % |
| **COPD** | 3.8 % | 4.0 % |
| **Asthma** | 11.2 % | 10.8 % |
| **Rheumatoid Arthritis** | 4.6 % | 5.0 % |

Comparison between all the participants attending the 7^th^ Survey of the Tromsø Study and the final sample included in this article. All the characteristics reported in this table were included in our analyses.
