# Supplementary figures and images for: Prevalence and clinical associations of wheezes and crackles in the general population: the Tromsø study
Source: BMC Pulm Med. 2019 Sep 11;19:173. doi: 10.1186/s12890-019-0928-1 (PMC6739986; doi:10.1186/s12890-019-0928-1)

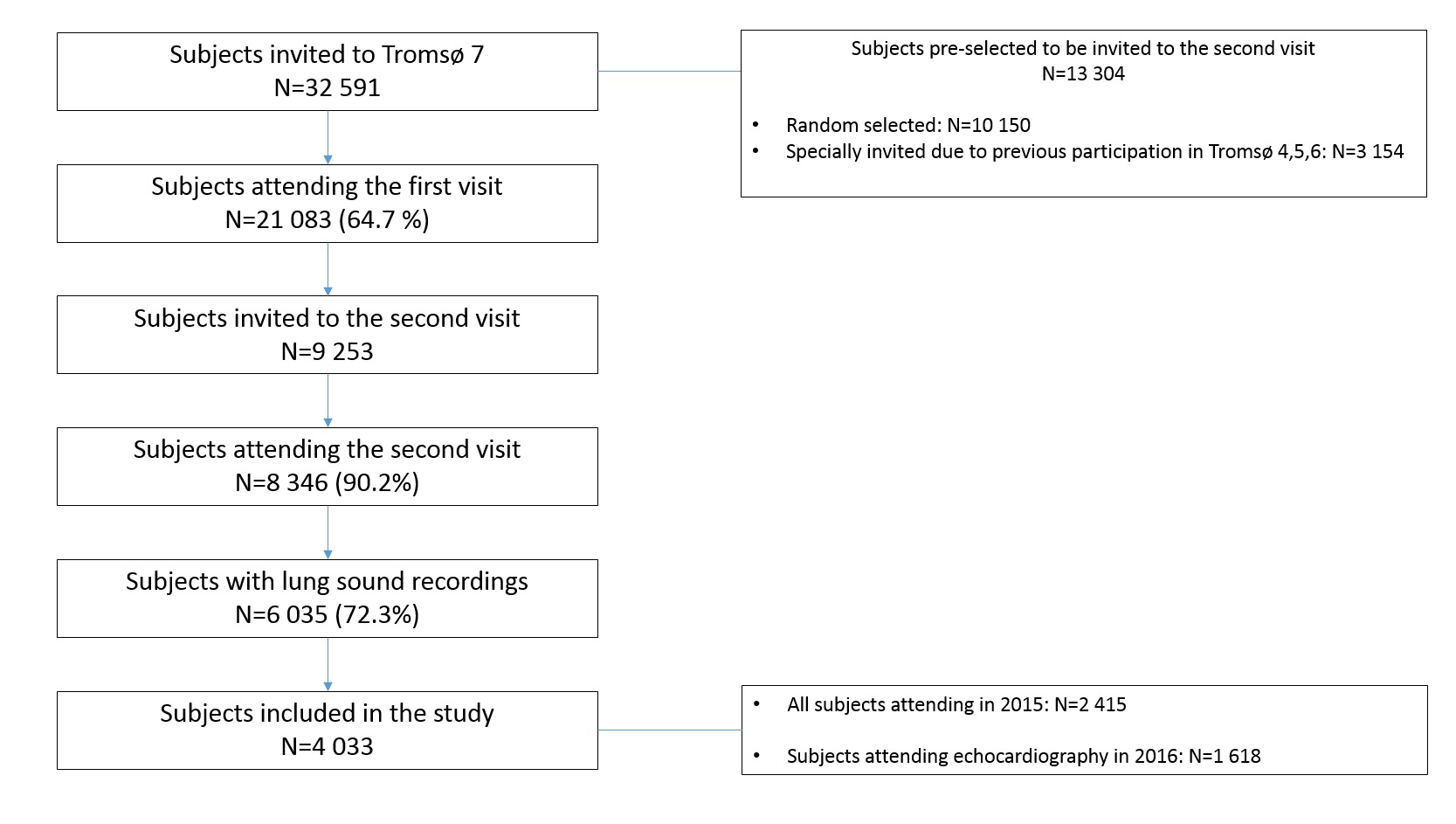

Supplement: Supplementary file 2 — Figure S1. Flow diagram of the participants included in our analyses. (JPG 292 kb) [file 12890_2019_928_MOESM2_ESM.jpg]
