# Supplementary material for: Prevalence and clinical associations of wheezes and crackles in the general population: the Tromsø study
Source: BMC Pulm Med. 2019 Sep 11;19:173. doi: 10.1186/s12890-019-0928-1 (PMC6739986; doi:10.1186/s12890-019-0928-1)
Supplement: Supplementary file 3 — Figure S2. Spirogram and clinical information of four participants with presence of adventitious lung sounds in the recordings. (PPTX 2605 kb) [file 12890_2019_928_MOESM3_ESM.pptx]

## Slide 1
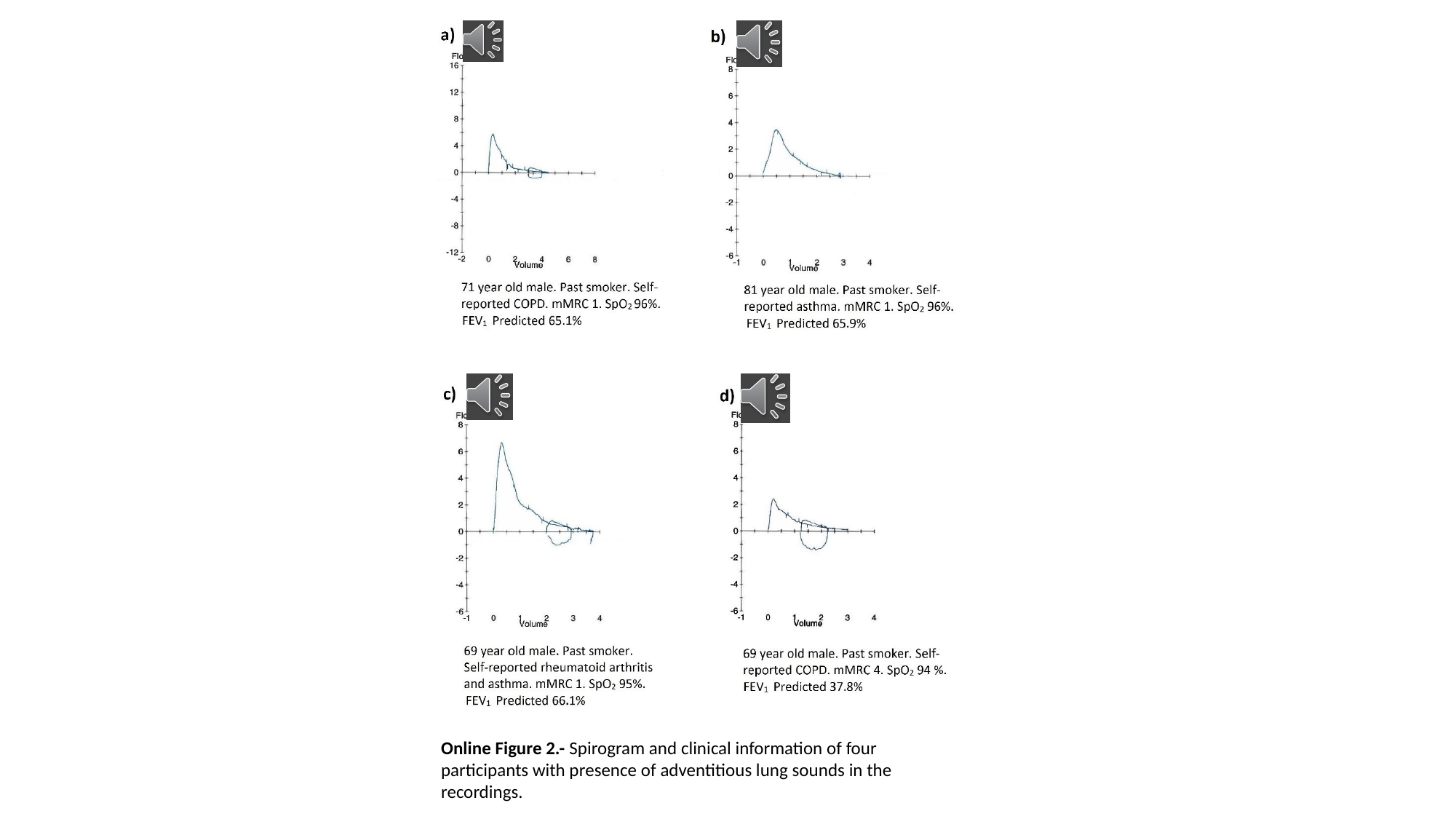

Online Figure 2.- Spirogram and clinical information of four participants with presence of adventitious lung sounds in the recordings.
